# Repetition-Related Reductions in Neural Activity during Emotional Simulations of Future Events

**DOI:** 10.1371/journal.pone.0138354

**Published:** 2015-09-21

**Authors:** Karl K. Szpunar, Helen G. Jing, Roland G. Benoit, Daniel L. Schacter

**Affiliations:** 1 University of Illinois at Chicago, Department of Psychology, Chicago, IL, United States of America; 2 Harvard University, Department of Psychology and Center for Brain Science, Cambridge, MA, United States of America; University of Tokyo, JAPAN

## Abstract

Simulations of future experiences are often emotionally arousing, and the tendency to repeatedly simulate negative future outcomes has been identified as a predictor of the onset of symptoms of anxiety. Nonetheless, next to nothing is known about how the healthy human brain processes repeated simulations of emotional future events. In this study, we present a paradigm that can be used to study repeated simulations of the emotional future in a manner that overcomes phenomenological confounds between positive and negative events. The results show that pulvinar nucleus and orbitofrontal cortex respectively demonstrate selective reductions in neural activity in response to frequently as compared to infrequently repeated simulations of negative and positive future events. Implications for research on repeated simulations of the emotional future in both non-clinical and clinical populations are discussed.

## Introduction

A large number of recent studies have examined the neural and cognitive processes associated with imagining or simulating possible future experiences [1,2,3]. An important characteristic of future simulations is that they are often emotionally arousing: recent evidence indicates that approximately two-thirds of everyday future thoughts are positively or negatively charged [4]. Repeatedly simulating negative future experiences can increase the subjective plausibility of such experiences [5], and both the vividness and likelihood of negative future experiences are heightened in patients with anxiety disorders [6]. Indeed, individual variation in the amount of repetitive negative thought about the future represents a known predictor for the development of diagnosable symptoms of anxiety [7,8]. Nonetheless, next to nothing is currently known about how the human brain processes repeated simulations of emotional future events. For instance, although various studies have examined the neural substrates of positive and negative simulations [9,10,11,12], they have not assessed how the observed pattern could be accounted for by known differences in positive and negative event simulations such as experienced vividness [5,13,14]. That is, activity differences favoring positive as compared to negative events may represent sensitivity to differences in emotional valence or vividness. Hence, using simple contrasts between positive and negative simulations as a starting point for understanding how the brain processes repeated simulations of emotional events may not be ideal.

As an alternative approach, we propose manipulating the frequency of simulation of emotional events. Recently, we showed that specific default network structures demonstrate repetition related reductions in neural activity to specific components of simulated events. For instance, in one experiment we asked participants to repeatedly simulate scenarios involving familiar people and places. Among other findings, we showed that regions of the default network commonly associated with representing information about locations, such as parahippcampal cortex showed repetition suppression when locations were repeated but not when people or scenarios tying people and locations together were repeated [15]. In the present study, we set out to manipulate the emotional content of future events (negative, positive, and neutral) and the frequency of exposure to those emotional events, thereby allowing us to determine which, if any, regions of the brain show selective reductions in neural responding to frequently as compared to infrequently repeated simulations of negative and positive events. This approach not only overcomes difficulties associated with cross-emotion comparisons, as we alluded to above, but it is also unbiased in terms of within-emotion comparisons. With regard to this latter point, prior work has shown that ratings associated with phenomenological characteristics such as detail increase with repetition [5], whereas our approach is primarily focused on illuminating regions that are preferentially responsive to infrequently as compared to frequently simulated events. We address the possible role of detail in mediating contrasts revealing greater activation for frequently as compared to infrequently simulated events in the discussion.

On the basis of prior work involving repeated simulations of non-emotional future events ([15,16,17], see also [18]), we expected infrequently as compared to frequently repeated simulations to more strongly evoke a distributed set of frontal, parietal, and temporal regions commonly associated with mental simulation of the future [2,3,19,20]. Although little is known about what regions of the brain are sensitive to repeated simulations of negative events, subcortical structures such as pulvinar nucleus and amygdala represent feasible candidates given their involvement in processing aversive stimuli in the context of threat and fear apprehension [21,22,23]. Notably, both structures possess significant interconnections with core regions implicated in mental simulation [24,25]. With regard to simulations of positive future events, prior research has demonstrated that medial orbitofrontal cortex is sensitive to repeated exposures of rewarding stimuli [26], suggesting that it may also be sensitive to repeated simulations of positive events. Finally, we expected increases in neural responding to repeated simulations of future events in ventral precuneus and inferior parietal lobule, as these regions have previously been associated with tracking frequency of event occurrence during mental simulation [15].

## Materials and Methods

### Participants

Participants were 26 right-handed adults with normal or corrected-to-normal vision and with no prior history of neurological or psychiatric impairment. 4 participants were excluded from data analysis (1 due to scanner error, 1 due to failure to understand instructions, and 2 due to excessive movement). The remaining 22 participants (18 female; mean age, 20.4 years) were included in all subsequent statistical analyses. The Committee of the Use of Human Subjects at Harvard University reviewed and approved the protocol and procedures carried out in this study. All participants subsequently provided informed written consent in accordance with this approved protocol.

### Stimulus collection and preparation

**Fig 1:**
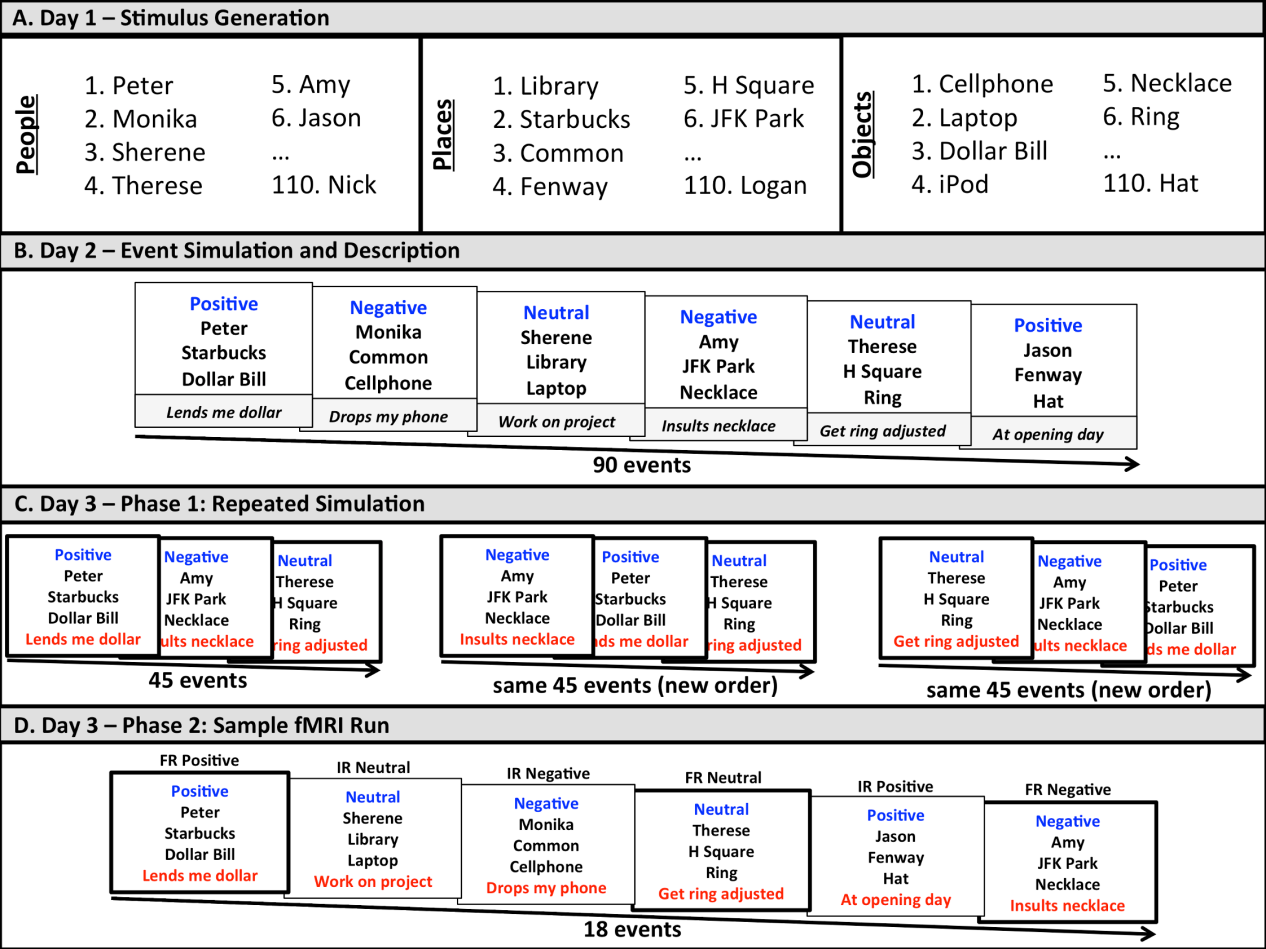
Illustrative diagram of repeated simulation paradigm. (A) During an initial visit, participants generated lists of 110 familiar people, 110 familiar locations, and 110 familiar objects that were later randomly re-organized to form 90 person-location-object triads that served as simulation cues for the study. (B) During a second visit, one week after the first visit, participants were required to simulate future events in response to 90 person-location-object triads or simulation cues. Each cue was paired with a positive (30), negative (30), or neutral (30) emotion tag and participants were given 12.5 s to generate a future event that involved interacting with the specified person and object in the specified location that would make the participant feel in accordance with the specified emotion. After 12.5 s, participants were required to type a brief description of the event that would later help them to re-imagine the same event. (C). During a third visit, one day after the second visit, participants were required to re-simulate 45 of the 90 events (15 positive, 15 negative, and 15 neutral). These 45 events were each simulated three times, each time in a new random order. Each simulation cue was comprised of the person-location-object triad, the emotion tag, and the participant’s previously generated description of the event. Participants were given 12.5 s to re-simulate each event. (D) During the third visit, and 10 minutes following re-simulation, participants were placed in the fMRI scanner and asked to imagine all 90 events (45 repeated, 45 non-repeated) one more time. The events were simulated over the course of 5 scans [18 events per scan; 6 *negative frequently repeated (FR)*, 6 *positive FR*, 6 *neutral FR*, 6 *negative infrequently repeated (IR)*, 6 *positive IR*, and 6 *neutral IR* trials per scan). Each trial involved a 30 s time window that comprised a preparatory ‘simulate’ cue (2.5 sec), a simulation cue (emotion tag, person-location-object triad, event description; 12.5 s), and memory, subjective detail, and subjective plausibility ratings (5 s each; see Methods for additional details).

During a first visit, participants were asked to provide lists of 110 familiar people (first and last names; participants were encouraged to list familiar individuals from their Facebook, email, or phone contacts), 110 familiar locations (locations were instructed to be as specific as possible; “Harvard Square” was too general, whereas “Starbucks in Harvard Square” was acceptable), and 110 familiar objects (portable, location-neutral objects that could fit easily into a backpack) (see Fig 1A). For each participant, we selected 93 people, 93 locations, and 93 objects that met the above criteria. To ensure that all simulations would be of novel events and not merely variations of recalled experiences, these lists were randomly combined to create 90 person-location-object triads that served as simulation cues in the subsequent session. Three additional person-location-object cues were used for the purpose of practice trials. The first visit lasted approximately 2 hrs.

### Initial event generation and re-simulation

Participants returned 1 week later for approximately 1 hour to simulate 30 positive, 30 negative, and 30 neutral events that were presented in random order. On each of the 90 trials, participants were shown a simulation cue (i.e., a person-location-object triad) that was accompanied by either a positive, negative, or neutral emotion tag (i.e., the word ‘positive’, ‘negative’, or ‘neutral’) [14] presented in blue font to help participants quickly identify the emotion associated with the to-be-simulated event (see Fig 1B). Participants were allotted 12.5 s to imagine a positive, negative, or neutral future scenario that would take place within the next 5 years, and that would involve interacting with the specified person and the specified object within the specified location. As an example, one participant who was cued to simulate a negative event involving a friend, a local coffee shop, and their cell phone imagined encountering the friend at the grocery store at which point the friend requested to borrow the phone and promptly dropped and broke the phone. This hypothetical experience evoked a negative response from the participant. Had the event been paired with a positive emotional tag the participant could have imagined that the phone exchange went smoothly and that the friend expressed gratitude for the kind gesture. At the end of each trial, participants were prompted to type a brief (a few words) summary description of the imagined experience. Participants first completed three practice trials with the experimenter to ensure that they understood all instructions, and subsequently reported that all event simulations were novel (i.e., participants had not previously experienced or thought about these events). Materials were presented with E-Prime software Version 1.0 (Psychology Software Tools, Pittsburgh, PA) on a Dell desktop computer, and participants used a keyboard to type their summary descriptions. Following this session, the descriptions were incorporated into the simulation cues for presentation on the following day.

One the following day, participants returned for 1.5 hours to re-simulate half of the previously generated events (i.e., 15 negative, 15 positive, and 15 neutral) three times each in random order (see Fig 1C). Each of the 135 trials was displayed on the screen as a person-location-object triad paired with a negative, positive, or neutral emotion tag, and was additionally presented along with the participants’ summary descriptions (presented in red font). The large number of trials associated with this re-simulation phase required this session to be conducted outside of the scanner. Note that the purpose of the summary descriptions was to minimize the need for participants to spend time trying to remember what they had previously imagined in relation to these simulation cues. Participants were given 12.5 s to re-simulate each event as they had the day before without generating additional details. To ensure that participants would remain focused on the simulation task, they were told that they would later answer some questions about what they experienced throughout the session. Although time did not permit us to collect explicit descriptions across repeated simulations, participants subsequently reported that the summary descriptions enabled them to follow instructions (i.e., simulate the same event across repeated trials).

### Event simulation in fMRI scanner

After a 10-minute break following re-simulation of half of the events, participants were placed in the fMRI scanner. They were asked to re-simulate all 90 of the originally generated events (30 negative, 30 positive, and 30 neutral), so that these future experiences were simulated for either the first or fourth time that day (see Fig 1D). It is important to note that although participants simulated distinct sets of events once or four times on the day of scanning, they had simulated these events once before on the previous day during the original generation of event descriptions. Thus, these events were simulated twice or five times in total.

Participants simulated events in an event-related manner (i.e., events were interleaved with a number of different fixation intervals; see below) across five separate scans. Each event was presented in the context of a trial that lasted 30 s, and consisted of the following sequence: (1) the word ‘simulate’ presented in the center of the computer screen for 2.5 s, alerting participants that they were about to simulate a future event; (2) a simulation cue (i.e., an emotion tag presented above a person-location-object triad, along with the participants brief description of the event) presented for 12.5 s, indicating the specific future event participants were to simulate (it was emphasized to participants to only use this time to imagine the events in as much detail as possible); (3) the question “imagined earlier?” presented above a 2-point rating scale for 5 s, alerting participants to rate, with a button press, whether or not they had imagined the future event at the beginning of the session before entering the scanner (1 = yes; 2 = no); (4) the question “how detailed?” presented above a 5-point rating scale for 5 s, alerting participants to rate how detailed their simulation of the future event was (1 = low detail; 5 = high detail); and (5) the question “how plausible?” presented above a 5-point rating scale for 5 s, alerting participants to rate how plausible their simulation of the future event was (1 = very implausible; 5 = very plausible). The order of the detail and plausibility questions was counterbalanced across participants. The purpose of the phenomenological ratings was to ensure that participants were able to distinguish between repeated and non-repeated simulations (memory rating), and that repeated simulation had the predicted effects of participants’ subjective experiences (detail and plausibility ratings) [5]. The presentation of each trial was randomly interleaved with 12.5 s, 15 s, or 17.5 s of fixation, so as to introduce temporal jitter into the experimental design and thereby allow for event-related analyses. Our use of long jitters was meant to ward off task fatigue that might have been experienced from the relatively long task trials (30 s total). Post-experiment interviews indicated that participants were able to focus their attention on simulating events during the entire 12.5 s time window allotted to simulating events, and to subsequently turn their attention to making phenomenological ratings with ease.

The 90 events were presented across a series of five scans. During each scan, participants were presented with and simulated a future event in response to 18 simulation cues (see Fig 1D). Six of the simulation cues in each scan referred to *negative* events, six to *positive* events, and six to *neutral* events. In addition, nine of the simulation cues in each scan referred to events that had been simulated four times before entering the scanner [once to generate a summary description and three times immediately before entering the scanner; *frequently repeated* (*FR*) events] and nine referred to events that had only been simulated once before entering the scanner [once to generate a summary description; *infrequently repeated* (*IR*) events]. Hence, each scan included three instances of each of the following six trial types: *negative FR* trials, *positive FR* trials, *neutral FR* trials, *negative IR* trials, *positive IR* trials, and *neutral IR* trials. The 18 simulation cues in each scan were presented in random order. Each scan lasted 14 min and 10 s.

### Data acquisition and analysis

#### fMRI acquisition

Imaging was conducted on a 3T Siemens Magnetom TimTrio Scanner, equipped with a 12-channel head coil. A laptop computer running E-Prime software controlled the stimulus display that was projected (via an LCD projector) onto a screen placed at the head of the bore. Participants viewed the screen through a mirror fastened to the head coil. Cushions were used to minimize head movement and earplugs were used to dampen scanner noise. Participants made responses using a button box placed by their right hand.

Anatomical images were acquired using a high-resolution three-dimensional magnetization-prepared rapid gradient echo sequence (MPRAGE; 176 sagittal slices, echo time [TE] = 1.64 ms, repetition time [TR] = 2530 ms, flip angle = 7 degrees, voxel size = 1 x 1 x 1 mm). Functional images (340 scans including simulation cues) were collected using a T2* gradient echo, echo-planar imaging (EPI) sequence sensitive to blood oxygen level-dependent (BOLD) contrast (TR = 2500 ms, TE = 30 ms, flip angle = 90 degrees, 3 x 3-mm in-plane resolution). Whole-brain coverage was obtained with 39 contiguous slices, acquired parallel to the anterior-posterior commissure plane (3-mm slice thickness, 0.5-mm skip between slices).

#### Imaging analyses

Imaging data acquired during functional scans were preprocessed and statistically evaluated using SPM8 (Wellcome Department of Imaging Neuroscience, London, UK). First, these data were preprocessed to remove sources of noise and artifact. The first four volumes (10 s) of each scan were excluded from analyses to account for T1 saturation effects. Preprocessing included the following: (1) slice-time correction to correct for differences in acquisition time between slices for each whole brain volume, (2) realignment within and across runs to correct for head movement, (3) spatial normalization to the Montreal Neurological Institute (MNI) template (resampled at 2 x 2 x 2 mm voxels), and (4) spatial smoothing (8-mm full-width at half maximum [FWHM]) using a Gaussian kernel.

Preprocessed data were analyzed using the general linear model. For each participant (i.e., fixed effects models), the BOLD response to each trial type (i.e., *negative FR* trials, *positive FR* trials, *neutral FR* trials, *negative IR* trials, *positive IR* trials, and *neutral IR* trials) was modeled using SPM8’s canonical hemodynamic response function over a 15 s time window (i.e., an epoch) that immediately followed trial onset. Because the only difference across trials over the first 15 s was the type of event that was being simulated (e.g., *IR* trials versus *FR* trials), it was assumed that statistical differences emerging as a result of contrasts between trial types could be safely attributed to differences in the makeup of events rather than to the processing of the initial ‘simulate’ cue [13]. An effect of no interest was used to code the 15 s time window that followed each simulation cue and that included the three phenomenological ratings (i.e., memory, detail, and plausibility).

The results of the fixed effects analyses were moved forward to a group-level (i.e., random effects) analysis. Within the context of a Two-Way Analysis of Variance, these analyses involved planned contrasts across the six trial types designed to identify regions showing activity reductions for simulations of future events in general (i.e., activity during a *IR* trial is greater than activity during a *FR* trial), and also regions that showed selective activity reductions for repeated simulations of negative, positive, and neutral events. The underlying logic of each of the planned contrasts was as follows: (1) Regions showing activity reductions for repeated simulations of **future events** in general (*IR* trials > *FR* trials); (2) Regions showing selective activity reductions for repeated simulations of **negative future events** [*negative IR* trials > *negative FR* trials exclusively masked with *positive + neutral IR* trials > *positive + neutral FR* trials (i.e., a mask isolating regions that showed activity reductions to repeated simulations of positive and neutral events)]; (3) Regions showing selective activity reductions for repeated simulations of **positive future events** [*positive IR* trials > *positive FR* trials exclusively masked with *negative + neutral IR* trials > *negative + neutral FR* trials (i.e., a mask isolating regions that showed activity reductions to repeated simulations of negative and neutral events)]; and (4) Regions showing selective activity reductions for repeated simulations of **neutral future events** [*neutral IR* trials > *neutral FR* trials exclusively masked with *positive + negative IR* trials > *positive + negative FR* trials (i.e., a mask isolating regions that showed activity reductions to repeated simulations of positive and negative events)]. The masked contrasts allowed us to assess whether specific regions were particularly sensitive to repeated simulations of a given emotion in a manner that avoids possible phenomenological confounds (see Behavioral Results; for a similar approach, see [13]). In each case, we also examined the reverse contrasts, so as to assess what regions of the brain were more responsive to frequently repeated as compared to infrequently repeated simulations of future events.

A whole brain FWE error correction (*p* < 0.05) was used to identify reliable patterns of activity. In addition, we also report activations in regions of the brain that were anticipated on the basis of relevant literature (see Introduction) and that survived a more lenient uncorrected threshold of p < 0.001 with 10 or more contiguous voxels (for a similar approach, see [27]). Importantly, we indicate in Tables 1–4 which peak activations survived a whole brain correction, and which peak activations only survived the more lenient threshold. We further indicate in Tables 1–4 which peak activations were anticipated on the basis of the existing literature. Regions only surviving the more lenient threshold and not anticipated on the basis of the existing literature (indicated as so in Tables 1–4) are nonetheless reported in the interest of facilitating future meta-analyses of mental simulation.

**Table 1:** Regions showing reductions in neural activity for repeated future events *IR* > *FR*.

Region	BA	x	y	z	t	Vox	Corr	Expect
Med. FG	25	-4	29	-15	6.46	308	*	Y
R. OFC	47	34	29	-9	5.9	522	**	Y
L. PCC	23	-5	-54	16	6.96	759	*	Y
L. ITG	19	-44	-60	-5	6.69	1268	*	Y
R. ITG	21	58	-9	-13	4.1	128	**	Y
L. Thalamus		-7	-13	10	3.94	97	**	N
R. Thalamus		23	-27	2	3.79	40	**	N
R. Thalamus		9	-9	15	3.49	19	**	N
L. MFG	9	-37	13	23	7.24	1358	*	N
R. MFG	9	41	12	30	6.31	539	*	N
Cuneus	18	24	-92	0	7.67	1191	*	N
R. Caudate		3	3	8	4.19	153	**	N
R. Cerebellum		13	-79	-29	8.18	1615	*	N
		6	-56	-39	7.15	214	*	N
L. Cerebellum		-31	-69	-40	3.87	17	**	N

Note. IR = infrequently repeated, FR = infrequently repeated, L = left, R = right, FG = frontal gyrus, OFC = orbitofrontal cortex, PCC = posterior cingulate cortex, ITG = inferior temporal gyrus, MFG = middle frontal gyrus, Vox = voxels, Corr = correction

* = p < .05 (family wise error)

** = p < .001 (uncorrected), Y = yes, N = no

**Table 2:** Regions showing selective reductions in neural activity. Regions showing selective reductions in neural activity for negative events [(*IR Negative* > *FR Negative*) exclusively masked with *Positive* and *Neutral* (see Methods)].

Region	BA	x	y	z	t	Vox	Corr	Expect	Fig 2B
L. Puvlinar		-3	-26	5	3.66	42	**	Y	left
R. Pulvinar		25	-25	2	3.87	42	**	Y	
Subcallosal Gyrus		-27	2	-9	3.76	43	**	N	

Note. IR = infrequently repeated, FR = frequently repeated, L = left, R = right, Vox = voxels, Corr = correction

** = p < .001 (uncorrected), Y = yes, N = no.

**Table 3:** Regions showing selective reductions in neural activity for positive events [(IR Positive > FR Positive) exclusively masked with Negative and Neutral (see Methods)].

Region	BA	x	y	z	t	Vox	Corr	Expect	Fig 2B
L. OFC	47	-28	15	-15	4.60	78	**	Y	right
R. OFC	47	29	23	-15	3.69	52	**	Y	
R. PreC Gyrus	9	43	5	35	3.94	44	**	N	
L. MFG	46	-41	26	16	3.55	24	**	N	
L. SFG	8	-19	22	42	3.79	50	**	N	
L. IPS	7	-22	-66	50	4.50	109	**	N	
L. MTG	37	-45	-46	1	4.09	40	**	N	
Thalamus		4	-21	2	3.55	27	**	N	
R. Cerebellum		27	-70	-39	4.43	20	**	N	
		4	-54	-20	4.18	31	**	N	

Note. IR = infrequently repeated, FR = frequently repeated, L = left, R = right, OFC = orbitofrontal cortex, PreC = precentral, MFG = middle frontal gyrus, SFG = superior frontal gyrus, IPS = inferior parietal sulcus, MTG = middle temporal gyrus, Vox = voxels, Corr = correction

** = p < .001 (uncorrected), Y = yes, N = no.

**Table 4:** Regions showing increases in neural activity for repeated future events *FR* > *IR*.

Region	BA	x	y	z	t	Vox	Corr	Expect
L. Ven. Precuneus	7	-12	-66	34	4.36	87	**	Y
R. Ven. Precuneus	7	12	-66	37	3.98	147	**	Y
L. IPL	40	-63	-30	36	4.32	289	**	Y
R. IPL	40	63	-37	33	5.67	815	**	Y
R. MFG	6	20	10	60	4.38	62	**	N
R. MFG	9	31	38	29	4.14	98	**	N
R. PreC Gyrus	44	45	7	7	3.7	56	**	N
L. Insula	13	-42	-4	6	3.82	84	**	N
Cingulate Gyrus	23	6	-21	25	5.32	495	**	N
Cingulate Gyrus	31	10	-29	42	3.76	40	**	N
R. Caudate		23	-41	12	4.23	68	**	N

Note. FR = frequently repeated, IR = infrequently repeated, L = Left, R = Right, Ven. = Ventral, IPL = inferior parietal lobule, MFG = middle frontal gyrus, preC = precentral, Vox = voxels, Corr = correction

** = p < .001 (uncorrected), Y = yes, N = no.

Exclusive masking was used to identify voxels for which effects were not shared between two contrasts (e.g., regions that showed selective reductions in neural activity for repeated simulations of negative future events, and not to positive or neutral events). The exclusive mask was set at a threshold of *p* < 0.05, whereas the masked contrast was kept at a FWE-corrected threshold and reduced to a threshold of *p* < 0.001 in cases where no reliable effects at a whole-brain correction emerged; the resulting threshold of the exclusive masking procedure was the same as the masked contrast (see Tables 1–4 for details). In order to further illustrate the reliable interactions that arose from these analyses, MarsBaR Toolbox was used to extract percent signal change for each of the six trial types from regions that were identified as selectively sensitive to repetitions of negative, positive, and neutral events. Finally, the peak MNI coordinates of active regions were converted to Talairach space, and regions of activation were localized in reference to a standard stereotaxic atlas.

## Results

### Behavioral results

Memory performance scores (Hits-FA = .99) indicate that participants were able to easily discriminate between repeated and non-repeated simulations of future events, suggesting that they followed task instructions closely. As further indication of the effectiveness of our repeated simulation manipulation, 2 [repetition: frequently repeated (FR) vs. infrequently repeated (IR)] x 3 (emotion: positive, negative, and neutral) repeated measures ANOVAs demonstrated strong effects of repetition such that frequently repeated future events were rated as more detailed [*M* = 3.70 versus 3.03; *F* (1,21) = 40.83, *p* < 0.001] and plausible [*M* = 2.93 versus 2.60; *F* (1,21) = 31.06, *p* < 0.001] than infrequently repeated future events (see Table 5). As outlined in the introduction, these behavioral effects of repeated simulation would only be relevant to neural contrasts of yielding greater activation for frequently as compared to infrequently simulated events. Further supporting our approach to avoid directly comparing positive and negative events, and largely in line with extant literature, our behavioral analyses revealed effects of emotion on detail [*F*(2,42) = 5.24, *p* = 0.009] and plausibility [*F*(2,42) = 22.75, *p* < 0.001]. With regard to detail, positive events (*M* = 3.51) were rated as more detailed than negative (*M* = 3.34) and neutral (*M* = 3.26) events [*t*(21) = 2.16, *p* = 0.043 and *t*(21) = 3.42, *p* = 0.003, respectively]. Negative and neutral events did not differ in terms of detail (*t* < 1). With regard to plausibility, positive (M = 2.88) and neutral (M = 2.89) events were rated as more plausible than negative events (M = 2.53) [*t*(21) = 5.99, *p* < 0.001 and *t*(21) = 5.66, *p* < 0.001, respectively]. Positive and neutral events did not differ in terms of plausibility (t < 1). Finally, there were no interactions between repetition and emotion for any of the ratings (all *F*s < 1).

**Table 5:** Mean ratings for events as a function of emotion and repetition.

**Detail**	**Positive**	**Negative**	**Neutral**	**Total**
FR	3.83 (0.35)	3.66 (0.38)	3.62 (0.40)	3.70 (0.30)
IR	3.18 (0.51)	3.01 (0.45)	2.89 (0.51)	3.03 (0.39)
Total	3.51 (0.33)	3.34 (0.33)	3.26 (0.33)	
**Plausibility**	**Positive**	**Negative**	**Neutral**	**Total**
FR	3.04 (0.48)	2.67 (0.55)	3.08 (0.49)	2.93 (0.44)
IR	2.72 (0.58)	2.39 (0.46)	2.69 (0.48)	2.60 (0.45)
Total	2.88 (0.48)	2.53 (0.48)	2.89 (0.40)	

Note. FR = frequently repeated, IR = infrequently repeated, Standard deviations are presented in parentheses.

### Regions demonstrating activity reductions for repeated simulations of future events

**Fig 2:**
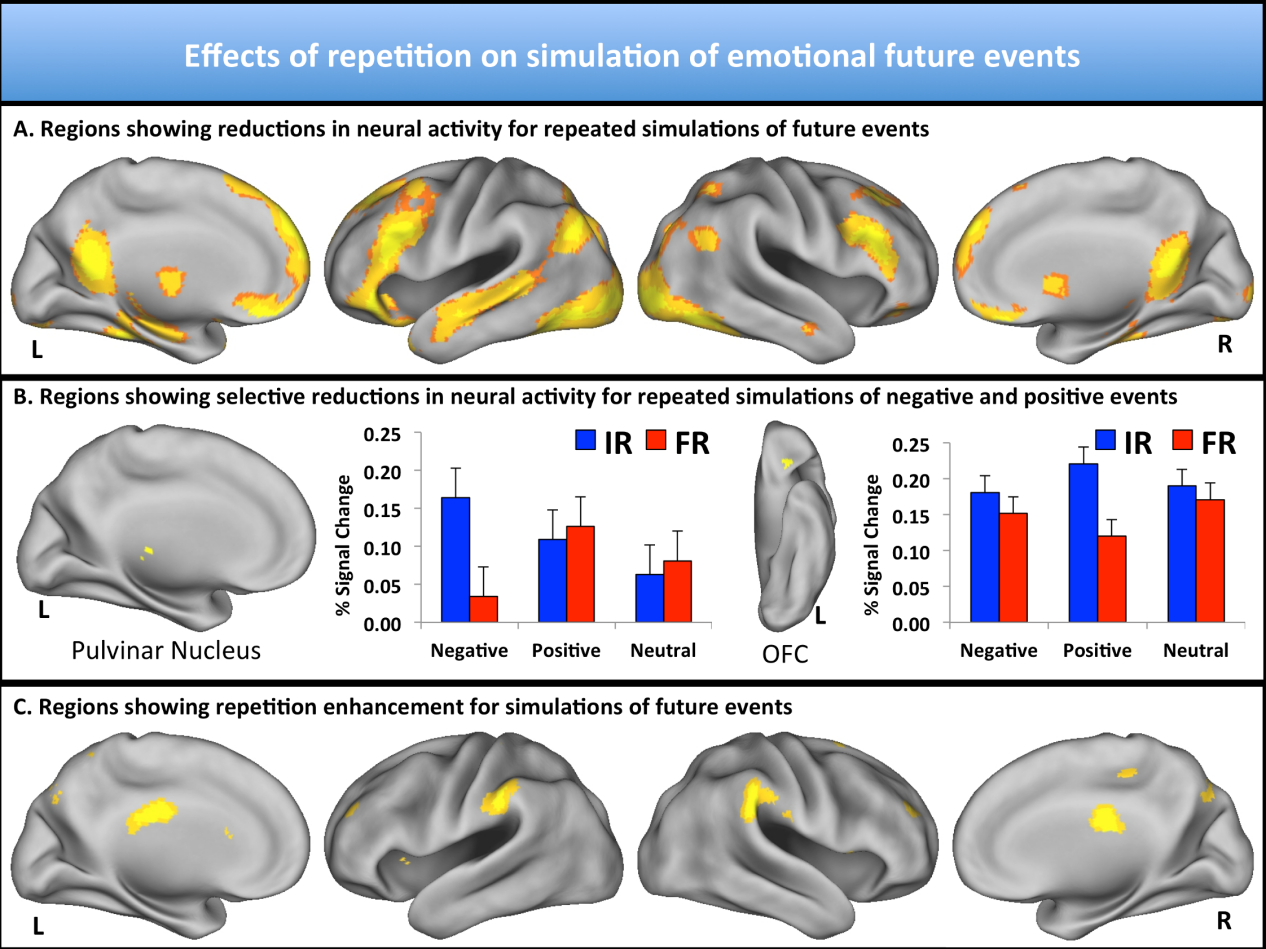
fMRI results. (A) Regions showing activity reductions [infrequently repeated (IR) > frequently repeated (FR) trials] to repeated simulations of the future included medial prefrontal cortex, posterior cingulate cortex, lateral temporal cortex (extending into lateral parietal cortex), thalamus, lateral prefrontal cortex, and cuneus. (B) Pulvinar nucleus (left) and orbitofrontal cortex (right) respectively showed selective reductions to repeated simulations of negative and positive future events. For illustrative purposes, bar graphs depict percent signal change for IR and FR negative, positive, and neutral trials. Error bars represent standard errors of the mean. (C) Regions showing activity increases to repeated simulations of the future (FR trials > IR trials) included bilateral ventral precuneus and bilateral inferior parietal lobule. All maps are shown as *p* < 0.001 (uncorrected) with minimum 10 voxels to help facilitate visualization.

Infrequent, relative to frequent, simulations of future events (i.e., *IR* trials > *FR* trials) recruited a distributed set of brain regions including medial prefrontal cortex, posterior cingulate cortex, and lateral temporal cortex (extending to lateral parietal cortex) that are commonly associated with the simulation of future events (see Fig 2A; Table 1; see [2,3,20]). In addition, infrequent relative to frequent simulations were associated with activity in lateral prefrontal cortex, cuneus, and a number of subcortical regions within the thalamus and cerebellum. While these repetition-related reductions in neural activity highlight cortical structures commonly associated with future event simulation, this general contrast did not reveal which regions were particularly sensitive to repeated simulations of negative, positive, or neutral events. Hence, we further compared and contrasted *IR* and *FR* trials that were specific to valence (as outlined in the Methods).

### Regions demonstrating selective activity reductions for repeated simulations of negative and positive events

Regions that were selectively sensitive to repeated simulations of negative events [i.e., showed activity reduction for negative events (*IR* negative > *FR* negative), but not for positive or neutral events] included the bilateral pulvinar nucleus and left subcallosal gyrus (Table 2). For illustrative purposes, Fig 2B demonstrates the pattern of activity across all six trial types in terms of percent signal change for left pulvinar nucleus. Conversely, and in line with predictions, regions that were selectively sensitive to repeated simulations of positive events [i.e., showed activity reduction for positive events (*IR* positive > *FR* positive), but not for negative or neutral events] included bilateral orbitofrontal cortex, along with a number of regions commonly associated with the control of attention, including inferior parietal and precentral cortices (Table 3). For illustrative purposes, Fig 2B demonstrates the pattern of activity across all six trial types in terms of percent signal change for left orbitofrontal cortex. No regions were selectively sensitive to repeated simulations of neutral events.

### Regions demonstrating activity increases for repeated simulations of future events

Replicating previous studies of repeated simulation of future events [15,16,17], various regions of the brain showed repetition enhancement or greater activity for frequently repeated as compared to infrequently repeated future event simulations. Among other regions, we found that bilateral ventral precuneus and inferior parietal lobule were especially responsive to frequently relative to infrequently repeated simulations (Fig 2C; see also Table 4). No interactions with emotion were observed. Given that frequently compared to infrequently repeated simulations were rated higher in detail and plausibility, we further sought to assess whether the difference in activation between the conditions is influenced by the differences in those two features. In order to do so, we calculated the average difference in detail and plausibility of frequently repeated trials versus infrequently repeated trials, separately for each participant. We then mean-centered these scores across participants, and separately entered the resulting values as covariates into the second-level analysis comparing frequently repeated and infrequently repeated events. These analyses revealed that activation in all but one of the regions emerging from the frequently repeated > infrequently repeated contrast did not interact with changes in detail or plausibility. On the other hand, repetition related increases in activity in caudate nucleus were found to be stronger for participants who showed greater increases in ratings of event plausibility with repetition (*p* < 0.001, 10 contiguous voxels).

## Discussion

The present study examined how the healthy human brain responds to repeated simulations of emotional simulations of future events. Using a novel repeated simulation paradigm, we found that infrequently relative to frequently repeated simulations of future events engaged a distributed set of frontal-parietal-temporal regions that have previously been demonstrated to support the ability to simulate future events [2,3,20,28,29,30]. Interestingly, we did not observe repetition related reductions in hippocampus [16,17]. One possibility is that, unlike prior studies, our manipulation varied the frequency of repeated simulation (and not the presence versus absence of repeated simulation), which may have minimized the role of constructive processes supported by hippocampus. Critically, our repeated simulation paradigm was able to further pinpoint specific regions that showed selective reductions in neural responding to negative and positive simulations of future events in a manner that avoided phenomenological confounds commonly observed between positive and negative events [5,14].

### Selective activity reductions to repeated simulations of negative future events

Of particular interest is the finding that the bilateral pulvinar nucleus showed selective reductions in neural responding to repeated simulations of negative, but not positive or neutral, future events. This pattern of data adds to the growing literature demonstrating the involvement of pulvinar nucleus in processing aversive stimuli [31,32,33]. To our knowledge, however, the present results represent one of the first demonstrations that this region may be involved in processing negative or aversive information that is not directly represented by a visual stimulus in the external environment (e.g., negative facial expressions), but rather conveyed by an imagined scenario (see also [22]). Moving forward, additional research will be needed to more specifically pinpoint the changes in cognitive/emotional processing that drive the selective reduction in neural responding of pulvinar nucleus to repeated simulations of negative future events. For instance, it is possible that repeated simulations of negative events are associated with reductions in the effort involved in generating the possible aversive consequences in relation to those events or perhaps reductions in the level of negative arousal or emotionality [34].

Finally, although some caution should be taken in interpreting our results as being localized to pulvinar nucleus, we note that the pulvinar is a relatively large subcortical structure and that it is the largest nucleus within the thalamus [35]. We also observed a similar pattern of results in the subcallosal gyrus. Both of these regions have further been associated with abnormally high levels of metabolic activity [36] and exaggerated resting state connectivity [37,38] in individuals diagnosed with major depression, who also demonstrated high symptoms of anxiety (for relevant discussion, see [39]). Though these observations are no more than suggestive, developing a clearer understanding of how regions in or near the pulvinar nucleus, and subcallosal cingulum, process repeated simulations of negative future events in both healthy and mood disordered populations may represent fruitful lines of future research.

### Selective activity reductions to repeated simulations of positive future events

We also documented that bilateral orbitofrontal cortex showed selective neural reductions to repeated simulations of positive, but not negative or neutral, future events. Although considerable prior work has demonstrated a role for orbitofrontal cortex in processing reward-related stimuli [26,40,41,42], our findings nonetheless add credence to more recent findings indicating that this region is involved in processing both real *and* imagined rewards [11,43]. Although appearing somewhat lateral, the orbitofrontal regions observed in our study fall within the range of previously reported peaks that associate orbitofrontal activity with reward processing (cf. [40]). Importantly, several regions associated with attentional control, including inferior parietal cortex and precentral gyrus, similarly demonstrated a selective decrease in neural responding to repeated simulations of positive, but not negative or neutral, future events. Although we did not anticipate this specific aspect of the results, the data may have implications for understanding the relation of orbitofrontal cortex to attention networks, and the role of their interactions in the affective biasing of attention towards positive stimuli [44,45].

### Activity increases to repeated simulations of future events

As with prior studies of future event simulation, bilateral ventral precuneus demonstrated repetition enhancement across repeated simulations of future events, irrespective of the valence associated with those events [15,16,17]. Importantly, resting-state functional connectivity analyses have indicated that this region is distinct from the default network [46,47,48], and that it forms part of a visual network that may sustain more basic memory processes. For instance, we have previously noted that the ventral precuneus is sensitive to the repetition of various stimuli, suggesting that this region of the brain may be involved in tracking frequency of exposure at some abstract level [15]. Indeed, a post-hoc analysis indicated that activity in this region was not associated with increases in event detail or plausibility. Moreover, our results further indicate that inferior parietal lobule may subserve a similar function. Unlike ventral precuneus, inferior parietal lobule only seems to show repetition enhancement when other people are involved in simulated events [15]. The repeated simulation paradigm used in the present study appears to be well suited to parsing the contributions of various cortical networks to mental simulation. Further research into how repetition related reductions and repetition enhancement are related to one another in the context of mental simulation represents an important avenue for future inquiry.

### Conclusions

To our knowledge, the present study represents the first systematic attempt to understand how the healthy human brain processes repeated simulations of emotional (i.e., positive and negative) future events. Given the frequency with which healthy human adults simulate future events on a daily basis [4] and the fact that repeated simulation of negative future events represents an important predictor for the onset of various mood and anxiety disorders [8], understanding the nature of repeated emotional simulations has potentially broad implications. We believe that the results of the present study can serve as a basis for more detailed investigations into the neural bases for internal representations of the emotional future.
